# Efficacy of Stereotactic Body Radiotherapy for Recurrent or Residual Hepatocellular Carcinoma after Transcatheter Arterial Chemoembolization

**DOI:** 10.1155/2018/5481909

**Published:** 2018-03-04

**Authors:** Erhua Yao, Jinghong Chen, Xiaofang Zhao, Yinyan Zheng, Xianheng Wu, Fei Han, Hecheng Huang, Ping Liang, Jianmin Liu, Fasheng Wu, Lianxing Lin

**Affiliations:** ^1^Department of Radiation Oncology, Affiliated Shantou Hospital of Sun Yat-sen University, Shantou, Guangdong 515031, China; ^2^Shantou Center for Disease Control and Prevention, Shantou, Guangdong 515041, China; ^3^Infectious Diseases Laboratory, Ruikang Hospital, Guangxi Traditional Chinese Medical University, Nanning, Guangxi Zhuang Autonomous Region 530001, China; ^4^Department of Preventive Health, Affiliated Shantou Hospital of Sun Yat-sen University, 114 Waima Road, Shantou, Guangdong 515031, China; ^5^Department of Medical Imaging, Affiliated Shantou Hospital of Sun Yat-sen University, Shantou, Guangdong 515031, China; ^6^Department of Radiation Oncology, Ruikang Hospital, Guangxi Traditional Chinese Medical University, Nanning, Guangxi Zhuang, China

## Abstract

**Aim:**

To evaluate the efficacy and toxicity of hypofractionated stereotactic body radiotherapy (SBRT) for patients with recurrent or residual hepatocellular carcinoma (HCC) after transcatheter arterial chemoembolization (TACE).

**Methods:**

Between June 2008 and July 2015, thirty-three patients with HCC were treated by SBRT. There were 63 lesions in 33 patients. A total dose of 39–45 Gy/3–5 fractions was delivered to the 70–80% isodose line.

**Results:**

Objective response rate (CR + PR) was 84.8% at 6 months. The overall survival rate was 87.9%, 75.8%, 57.6%, and 45.5% at 6, 12, 18, and 24 months, respectively. Median overall survival was 19 months. At 3 months, AFP decreased by more than 75% in 51.5% of patients (17/33). Overall survival was significantly different (*P* < 0.001) between the group of patients for whom AFP decreased more than 75% and the group for whom AFP decreased by less than 75%. The AFP-negative rate was 48.5% (16/33) after 6 months. Eight patients (24.2%) had grade 1-2 transient fatigue, and 11 patients (33.3%) had grade 1-2 gastrointestinal reactions within 1 month.

**Conclusion:**

SBRT is a promising noninvasive and palliative treatment with acceptable toxicity for recurrent or residual HCC after TACE.

## 1. Introduction

There are differences between China and the Western world regarding hepatocellular carcinoma in the following ways: (1) alcoholic cirrhosis was a common aetiology of liver cancer in Europe and the United States. In China, it was more common that hepatitis B virus infection gradually developed into liver cirrhosis, with liver cancer as an end stage disease. (2) Approximately 70% of HCC patients had elevated AFP in China, whereas only 30% of European and American patients had similar findings. (3) While sorafenib was the standard treatment for advanced and recurrent hepatocellular carcinoma in Europe and the United States, fewer patients received sorafenib in China not only because of less than 10% drug efficacy but also because the drug did not enter the healthcare system in most areas. Currently, TACE is a commonly used method to treat liver cancer. However, it has difficulty reaching tumours of over 5 cm with complete ischaemia or necrosis. The hepatic mass is supplied by two blood vessels, the hepatic artery and the portal vein. Therefore, even if the tumour artery is completely embolized with TACE, the portal vein may still provide blood to residual tumour cells, thus providing a source for future recurrence and metastasis [1]. Therefore, the purpose of this study is to evaluate the efficacy and toxicity of SBRT in patients with recurrent or residual HCC after TACE.

## 2. Materials and Methods

### 2.1. Patient Characteristics

This retrospective study included thirty-three patients with recurrent or residual HCC from the Radiotherapy Department of Ruikang Hospital of the Guangxi Traditional Chinese Medicine University who were treated with SBRT between June 2008 and July 2015. All patient medical records were carefully reviewed. Inclusion criteria were as follows: (1) diagnosed with HCC by pathology; (2) initial treatment by TACE for at least 3 months; (3) recurrent and residual diagnostic criteria: in HCC, after TACE in the CT/MR examination, it was found that the tumour disappeared, was not enhanced, or had subsequent reappearance or that the enhancement of the tumor was defined as recurrence or decreased but remained defined as residual; after TACE, lipiodol artifacts and CT/MR contrast agents are mixed together to determine the need for biopsy; (4) a history of liver cirrhosis derived from hepatitis B and, after formal review following antiviral therapy, a negative result for hepatitis B virus DNA (<10^3^ copies/mL); (5) AFP levels greater than 400 ng/mL before the first TACE; (6) class A liver function by Child-Pugh scoring; (7) KPS ≥ 70; (8) a volume of normal liver tissue > 700 mL; (9) no invasion or lymph node metastasis; and (10) three or fewer lesions. During treatment, patients who had liver tumour surgery or used targeted drugs or systemic chemotherapy were excluded.

The median age of 33 patients was 55 years (range: 42–75), and 75.8% were male. All patients had either recurrent (*n* = 12, 36.4%) or residual (*n* = 21, 63.6%) disease as determined by experienced radiation diagnostic physicians and radiologists. All patients underwent TACE 1–4 fraction. Cisplatin, Adriamycin, and/or mitomycin C were administered during TACE. Patient characteristics are presented in Table 1.

### 2.2. Radiation Therapy

The livers of all patients were implanted with 3-4 fiducials (99.9% pure gold particles) one week before the CT location scan. Fiducials localize to the tumour, within 2 cm, and no three gold fiducials can be coplanar. The tumour area was identified and outlined using a plain CT scan image as the basis to fuse CT/MR/DSA enhanced images. A plan was designed with 4D-CT treatment simulation using the CyberKnife® Robotic Radiosurgery System: X-sight Fiducials Tracking System (Accuray Incorporated, Sunnyvale, CA, USA). Target and critical structures (spinal cord, lung, heart, oesophagus, remaining healthy liver, stomach, intestine, and kidneys) were contoured. The GTV (gross target volume) was defined as a tumour visible on the CT/MR scan. The GTV was expanded by 3–5 mm to form the PTV (plan target volume). All patients were treated with a total dose of 39–45 Gy/3–5 fractions. The mean target volume was 128 cm^3^ (range: 36–236 cm^3^). Plans were devised so that the prescribed dose was the isodose line encompassing >96% of the PTV. No more than 3% of the PTV was to receive <94% of the prescribed dose. Patients were treated with the Accuray CyberKnife (1310 Chesapeake Terrace, Sunnyvale, CA 94089, USA). Dose constraints are shown in Table 2.

### 2.3. AFP

An index AFP level was recorded before TACE and before SBRT. AFP levels were also recorded at 3 months and 6 months after SBRT or at 1 month before death for those with a survival time less than 6 months.

### 2.4. Toxicity

Toxicity induced by SBRT was scored according to the NCI Common Terminology Criteria for Adverse Events (CTCAE) version 4.03. Radiation-induced liver disease (RILD) was defined as an anicteric elevation in alkaline phosphatase of at least 2-fold the upper normal level (classic RILD) or elevated transaminases of at least 5-fold (nonclassic RILD), without progressive disease (PD) and the development of nonmalignant ascites [2]. Acute toxicities were defined as those occurring within 90 days of SBRT. Late toxicities were defined as those occurring afterwards.

### 2.5. Statistical Methods

Survival rates were calculated from the date of SBRT. Kaplan-Meier survival analysis was used to estimate overall survival (OS). SPSS® software version 17.0 (IBM Corp., Armonk, NY) was used for statistical analyses. *P* < 0.05 was considered statistically significant.

## 3. Results

### 3.1. Tumour Local Control Rate

6 months after treatment, all patients underwent CT/MR examination. According to mRECIST criteria, there were 63 lesions in 33 patients. The degree of change was as follows: CR: 7 (18. 9%), PR: 33 (56. 9%), and PD: 14 (24. 2%). Objective response rate (CR + PR) was 84.8% (28/33) at 6 months.

### 3.2. Overall Survival Rate

**Figure 1:**
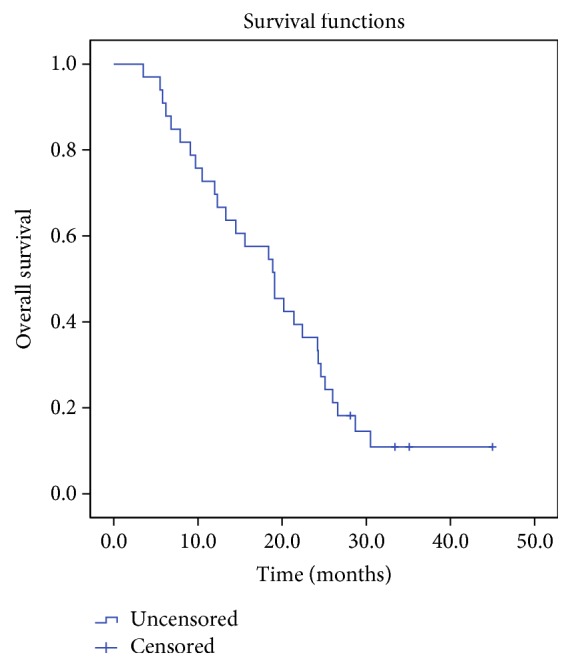
The Kaplan-Meier curve for overall survival.

The overall survival rates at 6, 12, 18, and 24 months were 87.9%, 75.8%, 57.6%, and 45.5%, respectively. Median overall survival was 19 months. The Kaplan-Meier curve for overall survival is presented in Figure 1.

### 3.3. AFP

**Figure 2:**
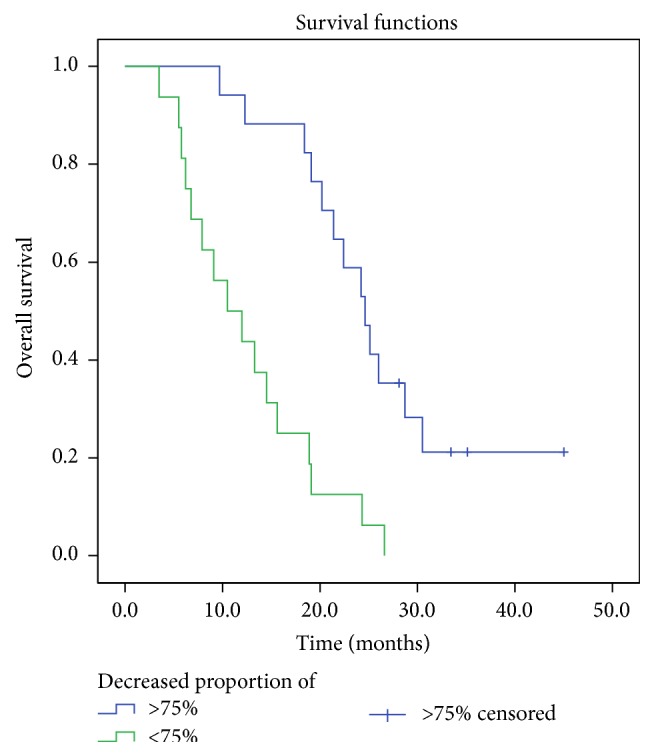
Kaplan-Meier survival curve for overall survival stratified by significant difference (*P* ≤ 0.001) between the group of patients for whom AFP decreased more than 75% and the group for whom AFP decreased by less than 75%.

The AFP index values of all patients were more than 400 ng/mL before TACE treatment. Those decreased at various rates after TACE. In the recurrent disease group, the AFP index of 4 patients fell to normal levels after TACE (chemiluminescence method: normal range is 0–4 ng/mL). However, the levels rose again after 3 months. The remaining patients had no reduction to normal levels. In the residual disease group, no patients had their AFP index decline to the normal range after TACE. At 3 months, AFP decreased by more than 75% in 51.5% of patients (17/33). Overall survival was significantly different (*P* ≤ 0.001) between the group of patients for whom AFP decreased more than 75% and the group for whom AFP decreased by less than 75%. The Kaplan-Meier curve for overall survival is presented in Figure 2.

### 3.4. Toxicity

8 patients (24.2%) had grade 1-2 transient fatigue, and 11 patients (33.3%) had grade 1-2 gastrointestinal reactions within 1 month. The symptoms were relieved or had disappeared after symptomatic and supportive treatment. No grade 3 or higher acute toxicities attributable to SBRT were found at 1 month (Table 3). 2 patients experienced liver failure toxicity at 3 months, and 1 patient was relieved after treatment. 1 patient experienced gastrointestinal haemorrhage at 6 months. 3 patients experienced anemia at 1 month and one of them turned worse at 6 months.

## 4. Discussion

Tumour tissues could not be completely eliminated through TACE. Previous studies reported that approximately only 22%–50% of the tumour tissue was totally destroyed as determined by pathology examination. The rate of recurrence and metastasis correlated with tumour size, type, scope, biological characteristics, collateral circulation, liver function, embolization, lipiodol dosage, and deposition [3].

There is no uniform suggestion for the treatment of residual cancer or recurrence of liver cancer. Surgery, TACE/TAE, radiation therapy, radiofrequency ablation, freezing, microwave, alcohol, and other methods have been reported in the literature [4–6]. The rate of recurrence after TACE/TAE is different from the rates following surgery, radiofrequency, freezing, or alcohol treatment. When the tumour cell undergoes necrosis after TACE/TAE of the hepatic artery, reembolization treatment is expected to be poor. It has been reported that TACE and PEI have been used in combination with TACE/TAE for the treatment of recurrence hepatocellular carcinoma. The authors believe that TACE combined with PEI treatment of liver cancer can make up for its respective deficiencies [7]. After TACE, the cancerous lesion was damaged, rendering it beneficial to inject more alcohol. Additionally, the embolization of the tumour in the arterial blood supply network was dispersed, which delayed the loss of alcohol. PEI causes coagulation and necrosis of hepatocellular carcinoma cells as well as the collateral circulation of the tumour and the blood supply of the portal vein. There had been reports [8] which compare the tumour control and SBRT combined with TACE for small and solitary HCC with TACE alone. Three hundred and sixty-five HCC patients who had solitary, ≤3 cm, and hypervascular nodule were treated with TACE. The results indicated that SBRT combined with TACE is a safe and effective modality for locoregional treatment of small solitary primary HCC and could be potentially a suitable option. Jacob et al. reported [9] that, in adjuvant stereotactic body radiotherapy following TACE in patients with nonresectable hepatocellular carcinoma tumours of ≥3 cm, overall survival was found to be significantly increased in the TACE + SBRT group compared with the TACE-only group (33 months and 20 months, respectively; *P* = 0.02). Su et al. similarly reported [10] the effectiveness of SBRT for patients with primary or recurrent small HCC who were unsuitable for surgical resection or local ablative therapy. The dose of 42–46 Gy in 3–5 fractions and 28–30 Gy in 1 fraction was prescribed. Local control rate at 1 year was 90.9%. OS at 1, 3, and 5 years was 94.1%, 73.5%, and 64.3%, respectively. PFS at 1, 3, and 5 years was 82.7%, 58.3%, and 36.4%, respectively. Another study [11] was carried out to retrospectively compare the outcome and evaluate the prognostic factors of SBRT alone or as an adjunct to transarterial embolization (TAE) or TACE in the treatment of HCC > 5 cm; SBRT combined with TAE/TACE may be an effective complementary treatment approach for HCC > 5 cm in diameter; BED10 ≥ 100 Gy and EQD2 ≥ 74 Gy should receive more attention when the SBRT plan is designed. Recently, a phase 2 study involving SBRT and optional transarterial chemoembolization (TACE) was conducted in patients with Child-Pugh grade A or B and underlying solitary HCC (greatest tumour dimension, ≤4 cm) who were unsuitable candidates for resection and radiofrequency ablation; the prescription dose was 35 to 40 grays in 5 fractions; the 3-year local control rate was 96.3%, the 3-year liver-related cause-specific survival rate was 72.5%, and the overall survival rate was 66.7%. Grade 3 laboratory abnormalities were observed in 6 patients, and 8 patients had Child-Pugh scores that worsened by 2 points. In conclusion, SBRT achieved high local control and overall survival with feasible toxicities for patients with solitary HCC, despite rather stringent conditions. SBRT can be effective against solitary HCC in treatment of naive, intrahepatic failure, residual disease, and recurrent settings, taking advantage of its distinctive characteristics [12, 13].

In our study, the overall survival rate was 87.9% (29/33), 75.8% (25/33), 57.6% (19/33), and 45.5% (15/33) at 6, 12, 18, and 24 months, respectively. Median overall survival was 19 months. Therefore, SBRT combined with TAE/TACE may be an effective complementary treatment approach.

Detection of serum alpha-fetoprotein (AFP) is used in clinical follow-up for high-risk population screening and cancer patients. Although 30% ~ 40% of patients have negative AFP detection in HCC of China [14], due to its characteristics of high specificity, it is still of significant value in clinical examination. In this study, the selected patients had AFP levels greater than 400 ng/mL before the first TACE treatment. Although AFP levels declined after TACE, they still remained higher than normal in recurrent and residual disease. The greater decline of AFP during the first 3 months of SBRT appeared to be associated with a better prognosis. The rate of AFP decline reflects the response of HCC therapy. At 3 months, 16 patients in whom AFP levels did not decrease up to 75% during the follow-up period were deceased, whereas 4 patients were still alive. Although hepatitis B can also lead to elevated AFP, formal antiviral therapy and resulting virus DNA negative status will result in a minimal influence by hepatitis on AFP levels. Additionally, the author observed that, in 4 patients, AFP levels fell to normal, were reelevated after TACE, and were reduced to normal again with subsequent SBRT; this was associated with longer survival (4 patients had median survival of 22 months).

Expanding the boundary outside the target area directly affects the control of tumours in radiotherapy. How far can liver cancer be confined to the liver in a subclinical tumour? A study on the range of foreign invasion of subclinical foci provided the basis for us to determine the imaging needs for the targeted tumour to evaluate the extent of expansion. After research, the tumour boundary was expanded 4 mm to include most of the area of invasion. If the tumour diameter was <5 cm, then 95% of tumour lesions could be treated by a 2 mm expansion [15]. These data are available for the range of the tumour target region (GTV) of the intrahepatic tumour target to the PTV. The extent of expansion met the tumour control needs. Most SBRT reports had similar findings.

In this study, 8 patients (24.2%) had grade 1-2 transient fatigue, and 11 patients (33.3%) had grade 1-2 gastrointestinal reactions within 1 month. The symptoms were relieved or eliminated after symptomatic and supportive treatment. No grade 3 or higher acute toxicities attributable to SBRT were found at 1 month. 2 patients experienced liver failure toxicity at 3 months, and 1 patient was relieved after treatment. 1 patient with enlargement of the spleen experienced gastrointestinal haemorrhage at 6 months. 3 patients experienced anemia at 1 month and one of them with long-term gastrointestinal reactions turned worse at 6 months. Most toxicities could be related to tolerance, which could be recovered after acceptance of treatment. In this study, patients with less adverse reactions may be associated with patients who have only selected Child-Pugh A class.

The liver has poor radiation tolerance. The occurrence of subacute radiation hepatitis is a standard for measurement. The total liver irradiation TD5/5 (minimum tolerance dose) is 25 Gy, and the whole liver irradiation level TD50/5 (maximum tolerated dose) is 40 Gy [16]. Therefore, a technique to maximise the amount of normal liver tissues and enhance the efficacy of tumour radiation is expected. With adjustable intensity modulated radiation therapy (IMRT) and stereotactic radiation therapy (SRT) development in the clinic, a growing number of HCC patients may receive different quantity and style of radiotherapy, and the therapeutic efficacy would not be merely palliative, especially for small HCC [17].

The major strengths of SBRT compared with conventional fractionated RT are as follows: (1) more intensified and accurate treatment of the tumour, (2) increased convenience for patients and technologists, and (3) minimal interference from delivery to the body itself [18]. SBRT is a promising noninvasive treatment with acceptable toxicity for recurrent or residual HCC patients after TACE. It is expected that quality of life and overall survival would benefit from SBRT for primary tumours. However, this needs to be warranted by a more prospective clinical study.

AFP was significantly elevated at the time of initial treatment. For patients receiving radiotherapy for recurrence of hepatocellular carcinoma, a 75% decline in AFP levels at 3 months may be a predictor of survival prognosis.

This study has several limitations. First, this was a retrospective single-center study. In this study, the short observation time, the small number of cases, and single-center study may affect the accuracy of the statistical test. Further prospective studies are needed to investigate the true effect of this novel treatment.

## 5. Conclusion

SBRT is a promising noninvasive and palliative treatment with acceptable toxicity for recurrent or residual HCC after TACE. And a >75% decline in AFP levels at 3 months may be a predictor of survival prognosis.

## Figures and Tables

**Table 1:** Patient characteristics.

Characteristics	Total *N* (%) or median (range)
Gender (*n*)	
Male	25 (75.8)
Female	8 (24.2)
Age (years)	55 (42–75)
Reason for uncontrolled disease (*n*)	
Recurrent	12 (36.4)
Residual	21 (63.6)
AFP before CyberKnife (ng/mL)	
>400	5 (15.2)
>200, <400	8 (24.2)
>50, <200	13 (39.4)
>4, <50	7 (21.2)
Clinical staging^*∗*^ (*n*)	
I	4 (12.1)
II	15 (45.5)
III	14 (42.4)

^*∗*^According to 2010 AJCC staging system.

**Table 2:** Dose constraints for critical structures (treatment in 3–5 fractions).

Critical structures	Dose constraints
Spinal cord	*V*18 < 1 mL
	max⁡20 Gy
Lungs (right + left)	*V*5 < 40%
	*V*10 < 20%
Heart	*V*20 < 15 cm^3^
	max⁡30 Gy
Oesophagus	*V*25 < 1 mL
Remaining healthy liver	*V*15 < 30%
	(*V*total − *V*15) > 700 cm^3^
Stomach	*V*25 < 1 mL
Intestines	*V*27 < 1 mL
Kidneys	*V*15 < 30%

**Table 3:** Toxicity after SBRT at 1, 3, and 6 months.

Toxicity (grade)	1 month		3 months		6 months	
	1, 2	3, 4	1, 2	3, 4	1, 2	3, 4
Transient fatigue	8	0	5	0	2	0
Gastrointestinal reactions	11	0	6	0	2	0
Anemia	3	0	2	0	2	1
Liver failure	0	0	0	2	1	1
Gastrointestinal hemorrhage	0	0	0	0	0	1

## Data Availability

The original anonymous dataset is available on request from the corresponding author at 35123948@qq.com.

## Additional Points

*Core Tip. *SBRT is a promising noninvasive and palliative treatment with acceptable toxicity for recurrent or residual HCC after TACE. There were 63 lesions in 33 patients, and a total dose of 39–45 Gy/3–5 fractions was delivered. Objective response rate was 84.8% at 6 months. The overall survival rate was 87.9%, 75.8%, 57.6%, and 45.5% at 6, 12, 18, and 24 months, respectively. Median overall survival was 19 months. In 3 months, AFP decreased by more than 75% in 51.5% of patients (17/33). Overall survival was significantly different (*P* ≤ 0.001) between the group of patients for whom AFP decreased more than 75% and the group for whom AFP decreased by less than 75%.
